# Effect of AFM Nanoindentation Loading Rate on the Characterization of Mechanical Properties of Vascular Endothelial Cell

**DOI:** 10.3390/mi11060562

**Published:** 2020-05-31

**Authors:** Lei Wang, Liguo Tian, Wenxiao Zhang, Zuobin Wang, Xianping Liu

**Affiliations:** 1 Center of Ultra-Precision Optoelectric Instrument Engineering, School of Instrumentation Science and Engineering, Harbin Institute of Technology, Harbin 150001, China; 2 International Research Center for Nano Handling and Manufacturing of China, Changchun University of Science and Technology, Changchun 130022, China; 5514tianliguo@163.com (L.T.); zhangwenx@cust.edu.cn (W.Z.); wangz@cust.edu.cn (Z.W.); 3 School of Engineering, University of Warwick, Coventry CV4 7AL, UK; X.Liu@warwick.ac.uk

**Keywords:** mechanical properties of cell, nanoindentation loading rate, atomic force microscopy, bEnd.3 cell, finite element analysis

## Abstract

Vascular endothelial cells form a barrier that blocks the delivery of drugs entering into brain tissue for central nervous system disease treatment. The mechanical responses of vascular endothelial cells play a key role in the progress of drugs passing through the blood–brain barrier. Although nanoindentation experiment by using AFM (Atomic Force Microscopy) has been widely used to investigate the mechanical properties of cells, the particular mechanism that determines the mechanical response of vascular endothelial cells is still poorly understood. In order to overcome this limitation, nanoindentation experiments were performed at different loading rates during the ramp stage to investigate the loading rate effect on the characterization of the mechanical properties of bEnd.3 cells (mouse brain endothelial cell line). Inverse finite element analysis was implemented to determine the mechanical properties of bEnd.3 cells. The loading rate effect appears to be more significant in short-term peak force than that in long-term force. A higher loading rate results in a larger value of elastic modulus of bEnd.3 cells, while some mechanical parameters show ambiguous regulation to the variation of indentation rate. This study provides new insights into the mechanical responses of vascular endothelial cells, which is important for a deeper understanding of the cell mechanobiological mechanism in the blood–brain barrier.

## 1. Introduction

The incidence of central nervous system (CNS) diseases represents a prevalent and heavy burden on the global health community, despite a significant improvement in the understanding of the pathological mechanisms [1]. The number of patients diagnosed as having Alzheimer’s disease is estimated as 45 million globally, and this amount is supposed to double every 20 years [2]. Although the demand for treatment of this disorder is urgent, only 7% of new drugs succeed in clinical development and reach the marketplace, while others have not yet been investigated or failed in clinical investigation due to their inability to cross the blood–brain barrier (BBB) and enter into the brain tissue [3]. The blood–brain barrier represents a unique interface that regulates the flowing of ions, nutrients, and other compounds entering into the brain tissue [4]. It is a double-edged sword in that the BBB plays a vital role in maintaining homeostasis and protecting the brain from pathogens invasion, however, the BBB blocks the transportation of drugs for the treatment of CNS diseases.

Vascular endothelial cells (VECs) exist around the blood vessel and comprise the interface between blood and the surrounding tissue [5]. These cells form the basis of the blood–brain- barrier, and the characterization of these cells is key to understanding pathologies and treating central nervous system disorders [6]. The investigation of VECs’ mechanical responses is still a challenge due to the difficult of obtaining reliable results among the wide range of mechanical parameters. Changes in the mechanical properties of VECs accompany a variation in the blood–brain barrier, which is due to these cells being the main barriers to the flow of ions, nutrients, and other nanoparticles meeting during the entering process into the brain tissue [7,8]. As a consequence, proper and accurate quantification of the mechanical properties of vascular endothelial cells is a key step to further understand the mechanism of blood–brain barrier, which has not been comprehensively understood until now.

A number of characterization techniques have been developed to precisely determine the mechanical properties of living cells, e.g., micropipette aspiration, microfluidic systems, microplates stretching or compressing, optical tweezer, magnetic tweezer and atomic force microscopy [9,10,11,12,13,14,15]. Micropipette, microplate and microfluidic systems characterize the global mechanical properties of single cell due to these techniques apply force on the whole cell. While techniques such as magnetic tweezer, optical tweezer and AFM measure the local mechanical properties at nanoscale with higher resolution. Among these techniques, AFM type scanning probe microscopes have the largest force range between pN and mN. The advantage of AFM technique is its ability to obtain the surface topography and mechanical properties simultaneously. Another benefit is different types of tips can be used in the experiment for different purposes. This is the reason why AFM based nanoindentation is widely used for the characterization of the mechanical properties of living cells.

A broad range of different cells were successfully investigated by AFM based nanoindentation. Liu et al. investigated the alteration of the morphology and biomechanical properties of living SMCC 7721 liver cancer cells treated with fullerenol (C_60_(OH)_24_) by using AFM, the effect of fullerenol on the cells’ elastic modulus were presented in their study [16]. Conventional Hertz contact model was used to fit the force-displacement curves obtained by AFM experiment in order to obtain the elastic modulus, which shows cell’s ability to recover its original shape when applied force released. A simple assumption was made in the analysis of experimental results that cell exhibits pure elastic property. Siamantouras et al. investigated the changes of cell stiffness and adhesion of human kidney tubule cell treated with TGF-β1, a significant increase was found in cell stiffness compared to control group without treatment with TGF-β1 [17]. Similarly, Hertz contact model was used to obtain the cell stiffness. This contact model is only valid under the assumptions including small strain and shallow indentation depth. It was reported that the values of one type of cell elasticity varied enormously in the literatures published by different research groups even using the same instrument [18]. This can be explained that cell has a complicated inner structure, including nucleus, cytoskeleton and organelles bathed in cytoplasm. All these elements contribute to the mechanical behavior of cells. Therefore, cells exhibit viscoelastic behavior with nonlinear phenomena. Nguyen et al. presented a methodology to study the nonlinear viscoelastic properties of breast cancer cells with AFM based nanoindentation [19]. The viscosity was observed with a hysteresis between loading and unloading force-displacement curves. Accurately and effectively quantification of the nonlinear mechanical properties of the cell is still a challenge over these years.

When it comes to vascular endothelial cells, Kang et al. investigated the mechanical properties of microvascular endothelial cells treated with TNF-α by using AFM and finite element analysis, a decrease in the shear modulus was found in all regions of endothelial cells [20]. The hyperelastic model was used to describe the mechanical behavior of endothelial cells. However, the viscosity properties were not considered in their study. It is important to note that viscous forces inherently regulate the interaction between nanoparticles and vascular endothelial cells during the entering process into the brain tissue. Although other technique such as microfluidic was used to investigate the effect of shear stress on vascular endothelial cell, the loading rate effect on viscosity behavior was not considered in previous studies [21]. The characterization of mechanical properties of vascular endothelial cell is key to understand the mechanism of blood–brain barrier, however there is no previous work on the characterization of the vascular endothelial cells using a comprehensive model considering multiple-mechanical behaviors.

The mechanical properties of vascular endothelial cells are not only related to their own physiology, but also to their interactions with nanoparticles which may cross the blood–brain- barrier to enter the brain tissue [22,23]. The variation of the mechanical properties of vascular endothelial cells are potentially linked to the effect of blood–brain barrier. While the mechanism that how these cells response to mechanical loading are still unclear. Therefore, more extensive and cutting-edge knowledge of the biomechanical properties of vascular endothelial cells is imperative to expand our understanding of the function of blood–brain barrier. In this work, a combined AFM nanoindentation experiment with inverse finite element analysis is used to investigate the nonlinear hyperelastic and viscoelastic properties of brain microvascular endothelial cell line bEnd.3. The nanoindentation protocol includes an indentation ramp under a certain loading rate with a stress relaxation period. Parameter analysis was performed to investigate the sensitivity of this model to a range of material parameters under different loading rates. The computation model with an optimization algorithm provides a framework to obtain the multiple mechanical properties with complicated cellular mechanism those cannot be obtained by experiments. Nanoindentation experiments with different loading rates in the ramp stage were performed to investigate the loading rate effect on the values of mechanical properties of bEnd.3 cells obtained by using this approach.

## 2. Materials and Methods

### 2.1. Cells Preparation

Nanoindentation experiment on cells were carried out by using mouse brain endothelial cell line bEnd.3, which was originally obtained from the cell bank at the Chinese Academy of Sciences. Cells were cultured in high glucose Dulbecco’s Modified Eagle’s Medium (DMEM, Gibco) supplemented with 10% fetal bovine serum (FBS, Hyclone), 100 U/mL penicillin (Solarbio), 100 mg/mL streptomycin (Solarbio) in an incubator with 5% CO_2_ kept at a constant temperature of 37 °C. The cells were passaged in about 2 days when they reached 80% of confluence. Cells used for AFM experiments were the fourth passages. Phosphate buffered saline (PBS) was used to wash the dishes and remove the dead cells before the AFM experiment.

### 2.2. AFM Experimentation

Nanoindentation experiments on bEnd.3 cells were carried out by using a home-made AFM system, which has successfully measured the adhesion force between the tip and cells as well as their morphology simultaneously [24]. The reliability of the results obtained by this system was validated in previous works when characterizing the mechanical properties and morphology of cells. The experiment was performed when cells were bathed in PBS solution at 20 °C. A silicon nitride probe (Bruker, MLCT) was used in this experiment and the nominal stiffness of selected cantilever was 0.07 N/m.

A triangle cantilever with tip was moved into the PBS solution and kept for half an hour for equilibration prior to the experiment. A 20× objective optical microscope was used to locate the accurate position of AFM tip over the central region (close to the nucleus) of the targeted bEnd.3 cell, which were placed on the substrate of glass. With the AFM tip moving downward and coming into contact with the surface of the cell, the deformation of the cantilever (z) and the vertical displacement of the probe ($d_{c}$) were recorded simultaneously. The indentation depth was determined from $d=z-d_{c}$ and the reaction force was F=k×d. Stress relaxation nanoindentation was carried out under a constant loading rate during ramp stage followed with a 10 s relaxation stage at the predefine depth of 1 μm. Four different loading rates at 0.1 μm/s, 0.5 μm/s, 1 μm/s, and 2 μm/s were selected to investigate the loading rate effect during the ramp stage in the experiment. Fifteen cells were selected for each loading rate.

### 2.3. Finite Element Model and Material Model for AFM Nanoindentation

**Figure 1:**
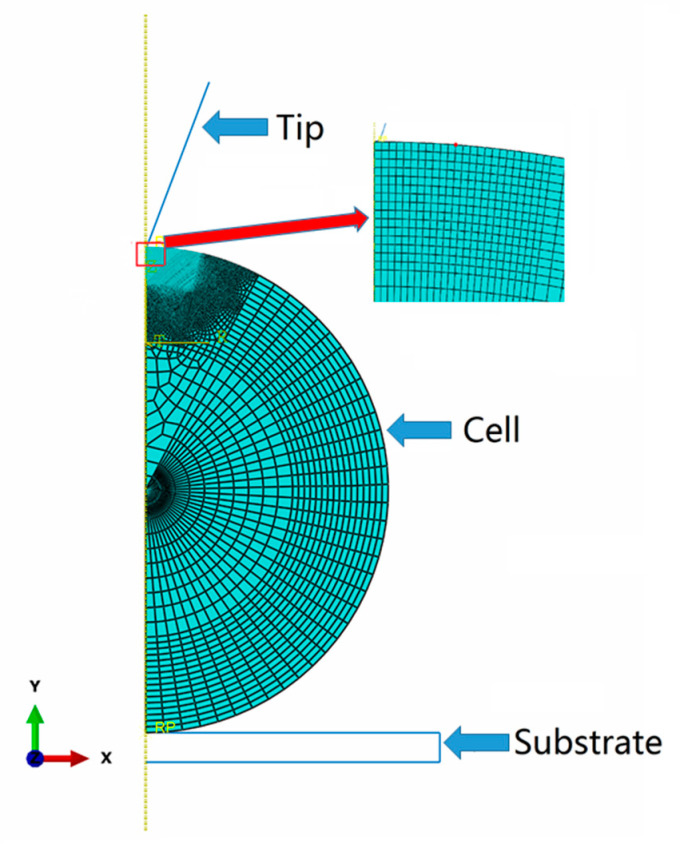
The finite element model of bEnd.3 cell subjected to nanoindentation.

The AFM nanoindentation experiment on bEnd.3 cell was simulated by using ABAQUS (version 6.14) software. The finite element model of tip, cell and substrate is shown in Figure 1. The indentation tip and glass substrate were modeled as rigid parts with no deformation due to the cell is much softer than the tip and glass substrate. The height of the cell was determined by the results of AFM measurements with an average value at 16.5 μm. Then cell was set in the simulation as an incompressible spherical sample with 8.25 μm in radius. The vertical indentation depth is 1 μm, which is much smaller than 10 percent of sample thickness. It is commonly believed that substrate effect can be neglected during the indentation process if the indentation depth is less than 10% of sample thickness [25].

Friction and thermal interaction between the tip, cell, and substrate were not considered in the simulation. The cell was simulated by using hybrid element CAX8H due to the sample was described by a model with hybrid properties. The contact area between the tip and the cell has a finer mesh than other regions. The convergence study of finite element analysis was performed and the average size in a fine mesh zone is about 1 nm. The nonlinear behavior of large deformation and time-dependent property were considered in this analysis, which is solved by using implicit time integration. The response force–time curve can be obtained from the output of the reaction force during the indentation ramp and stress relaxation stage.

In order to describe the mechanical behavior of bEnd.3 cell, a viscohyperelastic model was implemented in this study where the hyperelastic part was described by Neo-Hookean constitutive law. The strain energy function of a material described by Neo-Hookean model is listed below [26]:

(1) $$U=C_{10}(I_{1}-3)+\frac{1}{D_{1}}{\left(J-1\right)}^{2}$$

where U is strain energy density per volume unit and $I_{1}$ is first strain invariant. $C_{10}$ and $D_{1}$ are material parameters, which are given input to ABAQUS software. These two parameters correlate with shear modulus (μ) and compressibility modulus (k) as the following functions:

(2) $$C_{10}=\frac{\mu }{2}$$

(3) $$D_{1}=\frac{2}{\kappa }$$

The correlation between shear modulus (μ), compressibility modulus (k), and elastic modulus (E), Poisson’s ratio (υ) is shown as below:

(4) $$\mu =\frac{E}{2(1+\upsilon )}$$

(5) $$\kappa =\frac{E}{3(1-2\upsilon )}$$

Elastic modulus E and Poisson’s ratio υ can be calculated according to these equations. The viscoelastic part is described by an N-terms Prony series expansion of the dimensionless relaxation modulus [19]. The effective relaxation modulus is expressed in the function below:

(6) $$U_{r}=\mu \cdot [1-\sum_{k=1}^{N}g_{k}\cdot (1-e^{-t/\tau_{k}})]$$

where $g_{k}$ is the *k*th Prony constant (*k* = 1, 2, …, *N*), $\tau_{k}$ is the corresponding relaxation time constant, respectively. The Prony expansion is largely dominated by the first term in the series. Therefore, the number of term *N* is 1 in this work.

### 2.4. Parameters Sensitivity Analysis of Mechanical Parameters with Optimization

Parameter sensitivity analysis was performed to investigate the sensitivity of different material parameters, including material constant $C_{10}$, $D_{1}$, Prony expression constant $g_{1}$, time constant $\tau_{1}$, and indentation loading rate s during the ramp stage in AFM nanoindentation experiment. It is necessary to investigate the sensitivity of a material’s response to its mechanical properties in order to find an effective parameter iteration strategy. In addition, the results obtained from this analysis can be used as a guidance for inverse finite element analysis. One of these parameters varies with the value of other parameters were fixed, the obtained corresponding curves with relaxation response were recorded for comparison. The ranges of different material parameters in the nanoindentation stress relaxation experiment are shown in Table 1. In order to study the effect of loading rate on the stress relaxation response of cells when using this model, the parametric simulation study was carried out under different loading rate between 0.1 μm/s and 10 μm/s during the ramp stage. The relaxation time is 10 s with the tip kept still at the maximum depth.

**Figure 2:**
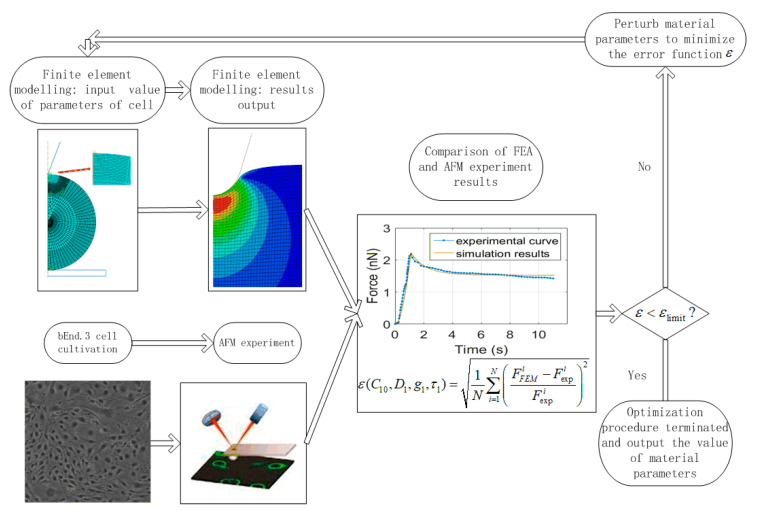
Flow chart of finite element analysis and AFM experiment combined with an optimization algorithm compiled in Matlab to obtain the mechanical properties of cells.

The mechanical properties of bEnd.3 cell were obtained by an optimization process combined with nanoindentation experiment and inverse finite element analysis until simulation curves matched those obtained from experiments when cells described by a viscohyperelastic model. The schematic of this procedure is shown in Figure 2. The differences between experimental results and finite element analysis is minimized by using an algorithm coded in Matlab software.

The detailed steps of the optimization procedure with inverse finite element analysis are provided as follows. For a given nanoindentation loading rate, the initial values of four parameters, $C_{10}$, $D_{1}$, $g_{1}$, $\tau_{1}$ are set as optimization variables in the pre-processing solver in ABAQUS. After initial simulation, the results are recorded and then simulated the force-time curve is compared with the corresponding curve obtained in experiment under the same loading rate. The differences between simulation results and experimental data are determined by minimizing the normalized mean squared error function:

(7) $$Min\left\{\varepsilon (C_{10},D_{1},g_{1},\tau_{1})=\sqrt{\frac{1}{N}\sum_{i=1}^{N}{\left(\frac{F_{FEM}^{i}-F_{\exp}^{i}}{F_{\exp}^{i}}\right)}^{2}}\right\}$$

in which the number of control points *N* corresponding to the number of load steps applied for the completion of finite element simulation of indentation with stress relaxation. $F_{FEM}^{i}$ and $F_{\exp}^{i}$ are the response force value of the *i*th load step computed by ABAQUS and corresponding measured in the experiment, respectively. The inverse analysis in the above equation is solved by using a multidimensional nonlinear minimization routine (fminsearch) in Matlab. The error ε is compared with a pre-defined limit $\varepsilon_{\mathrm{limit}}$ at 1% (0.01). If $\varepsilon >\varepsilon_{\mathrm{limit}}$, these parameters are perturbed in the subsequent iteratively until the convergence criterion is satisfied. When $\varepsilon <\varepsilon_{\mathrm{limit}}$, the optimization process terminates, and the optimized materials parameters of cell described by viscohyperelastic model are obtained under a given loading rate. This process was repeated for each nanoindentation loading rate applied in the experiment in order to investigate the loading rate effect on the mechanical properties. The large range of value for these parameters is selected to reduce the probability of missing the global optimum value.

## 3. Results

**Figure 3:**
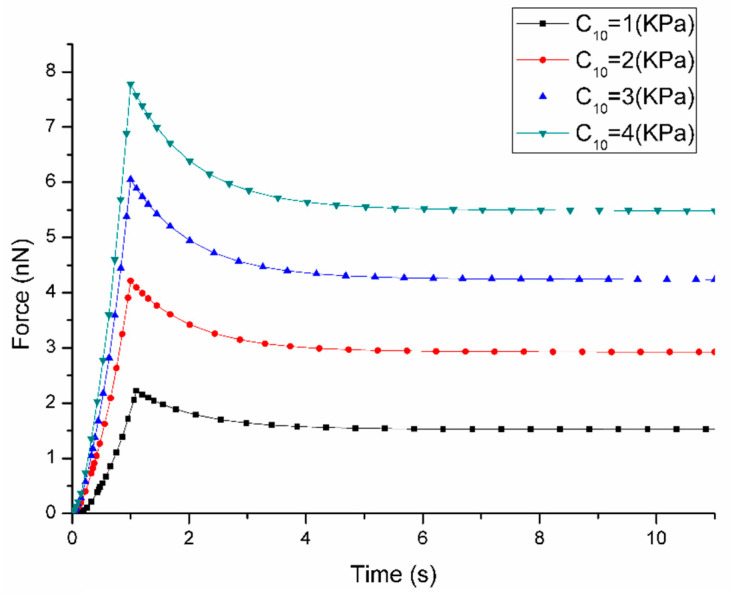
Sensitivity of Force-Time curves obtained from the variation of $C_{10}$ when the loading rate is 1 μm/s during ramp stage (the other parameters are fixed at: $D_{1}$ = 0.2 kPa^−1^, $g_{1}$ = 0.4, $\tau_{1}$ = 1 s).

**Figure 4:**
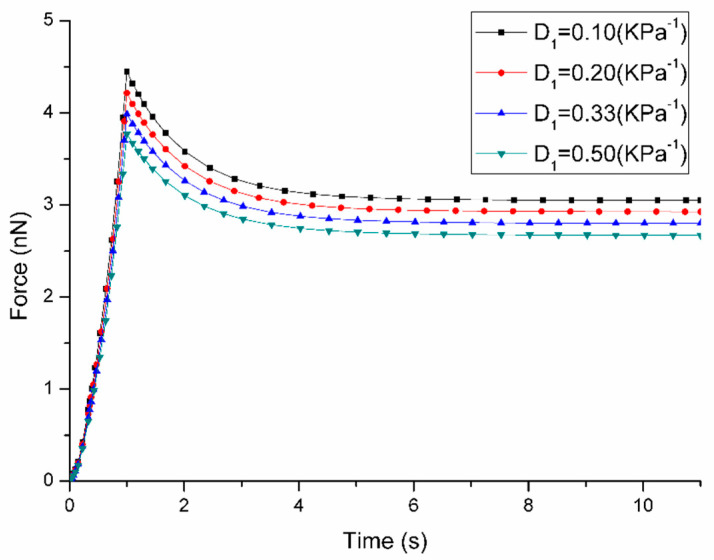
Sensitivity of Force-Time curves obtained from the variation of $D_{1}$ when the loading rate is 1 μm/s during ramp stage (the other parameters are fixed at: $C_{10}$ = 2 kPa, $g_{1}$ = 0.4, $\tau_{1}$ = 1 s).

**Figure 5:**
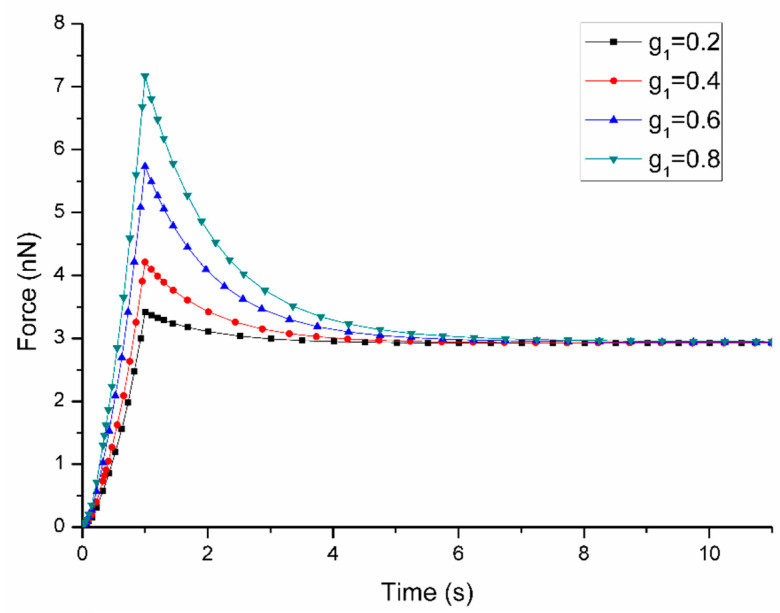
Sensitivity of Force-Time curves obtained from the variation of $g_{1}$ when the loading rate is 1 μm/s during ramp stage (the other parameters are fixed at: $C_{10}$ = 2 kPa, $D_{1}$ = 0.2 kPa^−1^, $\tau_{1}$ = 1 s).

**Figure 6:**
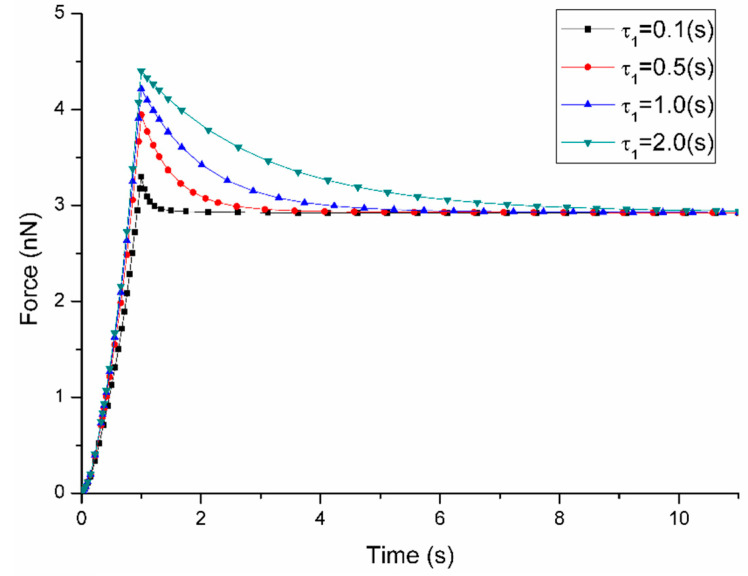
Sensitivity of Force-Time curves obtained from the variation of $\tau_{1}$ when the loading rate is 1 μm/s during ramp stage (the other parameters are fixed at: $C_{10}$ = 2 kPa, $D_{1}$ = 0.2 kPa^−1^, $g_{1}$ = 0.4).

The parameter sensitivity analysis demonstrated that these parameters show different effects on the short-term and long-term cell responses. The influence of parameter changes on the force-time curves are shown in Figure 3, Figure 4, Figure 5 and Figure 6. Figure 3 demonstrates that the variation of $C_{10}$ affects both short-term force response (peak force) and long-term force response of the cell. More specifically, when $C_{10}$ increases from 1 kPa to 4 kPa, the peak force rises from 2.22 nN to 7.77 nN with long-term force response also increasing from 1.52 nN to 5.48 nN. Although the variation of $D_{1}$ affects both short-term and long-term force responses of the cell, it shows a different trend that with the increasement of $D_{1}$ from 0.1 to 0.5 kPa ^−1^, the peak force reduces from 4.45 nN to 3.77 nN and long-term force decreases from 3.05 nN to 2.67 nN according to Figure 4. It is interesting to note that the variation of both $C_{10}$ and $D_{1}$ does not affect the duration when long-term force reached the equilibrium.

It is found in Figure 5 and Figure 6 that the variation of $g_{1}$ and $\tau_{1}$ only affect the short-term peak force of cell response while the long-term force responses are the same after approaching equilibrium. When the value of $g_{1}$ increases from 0.2 to 0.8, the short-term peak force climbs from 3.41 nN to 7.17 nN while long-term force response are constant at 2.93 nN. It is important to note that a larger value of $g_{1}$ results in a longer relaxation time to reach the equilibrium. A similar trend is also found in the parameter sensitivity analysis of the time constant $\tau_{1}$. As shown in Figure 6, an increase in the value of $\tau_{1}$ from 0.1 s to 2.0 s results in the increase of peak force from 3.31 nN to 4.41 nN with long-term force response at 2.93 nN. When the value of $\tau_{1}$ is smaller, it is faster to reach the equilibrium.

**Figure 7:**
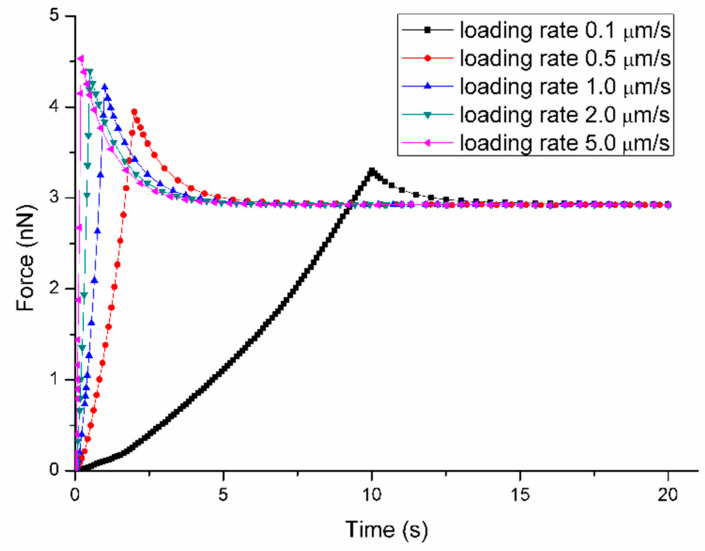
Sensitivity of Force-Time curves obtained from the variation of loading rate s when the mechanical parameters are fixed at: $C_{10}$ = 2 kPa, $D_{1}$ = 0.2 kPa^−1^, $g_{1}$ = 0.4, $\tau_{1}$ = 1 s.

In addition, the effect of loading rate during the ramp stage on the cell response was also performed in the sensitivity analysis. As shown in Figure 7, when the loading rate increases from 0.1 μm/s to 10 μm/s, the short-term peak force increases from 3.30 nN to 4.53 nN. Meanwhile the long-term force during the relaxation stage is stable at 2.93 nN. The time for force response to reach equilibrium is about 5 s for different loading rates.

**Figure 8:**
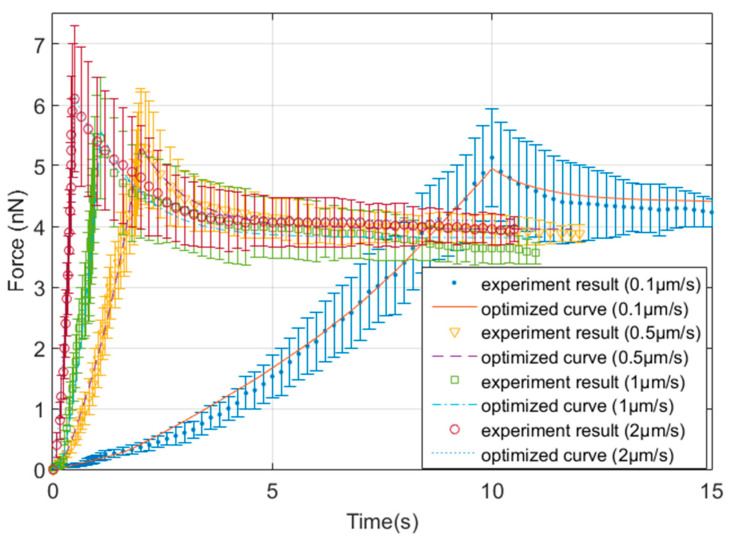
Comparison of mean experimental Force-Time curves and with optimized curves by inverse analysis under different indentation rate.

Figure 8 depicts force-time curves of each loading rate recorded in the experiment and the corresponding optimized curves obtained by the inverse analysis approach mentioned in Section 2.4. The effect of loading rate on the force curves in the experiment exhibits a similar trend to the sensitivity analysis in that a higher loading rate will result in a larger peak force. The values of mechanical properties determined by the optimized characterization procedure under different loading rates (0.1 μm/s to 2 μm/s) are listed in Table 2. The average values of mechanical parameters of bEnd.3 cells with the corresponding standard deviations are presented in this table. It is apparent from this table that the average value of $C_{10}$ and $D_{1}$ exhibit a clearer trend with the indentation rate than $g_{1}$ and $\tau_{1}$. When the indentation rate increases from 0.1 μm/s to 2 μm/s, the value of $C_{10}$ increases from 2.68 kPa to 3.32 kPa, while the value of $D_{1}$ reduces from 0.42 kPa^−1^ to 0.34 kPa^−1^. By implementing the relationship $E=6C_{10}$ derived for the Neo-Hookean model applied to incompressible materials, the range of elastic modulus of bEnd.3 cell is between 16.08 kPa and 19.92 kPa. The standard deviation is independent of loading rate, which indicates that the dispersion on these parameters is less sensitive to the loading rate than the biological variability of cells.

## 4. Discussion

The current study presents a novel approach that combined AFM experiment (ramp indentation followed by a stress relaxation process) with finite element simulation and optimization algorithms to obtain the mechanical properties of a single bEnd.3 cell. Although the viscoelastic properties of some mammal cells, such as oocytes and chondrocyte, have been investigated in previous works, no comprehensive study has been carried out on the viscohyperelastic behavior of vascular endothelial cells, which comprise the interface between blood and the surrounding tissue. A recent investigation of nanoparticles uptake by bEnd.3 cell only implements the simple elastic model to determine its mechanical properties [27]. The purpose of this study is to characterize the variation of force responses of bEnd.3 cell under different loading rates. Due to these force responses are one of the main factors regulating the blood–brain barrier, these detailed studies are the start point of any investigation of the blood–brain barrier, which is the physical barrier that regulates the ions, nutrients and other particles entering into the brain tissue. The variation of velocity of particle entering into the brain tissue may lead to significant changes in the reaction force. Significant differences in force responses are expected when using different load rate during the indentation ramp stage in the experiment. The results of this study indicate that the mechanical behavior of bEnd.3 cell can be well described by this viscohyperelastic model, which accounts for both nonlinear hyperelastic properties and time-dependent viscoelastic properties.

This approach is capable of obtaining the time independent part of cells’ response, which is driven by cell network structure, and the time-dependent part, which is driven by cytoplasm. The parametric analysis shows that the hyperelastic properties ($C_{10}$, $D_{1}$), which mainly arises from the cytoskeleton, contributes to short-term force responses of the cells. In contrast, cell viscoelastic properties ($g_{1}$, $\tau_{1}$) are shown to contribute to medium and long-term stress relaxation behaviors. An optimization algorithm minimizes the difference between AFM experiments and the corresponding simulation work for each loading rate. This provides a unique solution for bEnd.3 cells to obtain multi-parameters when described by a viscohyperelastic model.

The elastic modulus obtained in this study is higher than previous work done by Panzetta et al., who implemented polystyrene spheres (6 μm in diameter) as AFM tip to investigate the mechanical properties of bEnd.3 cell [27]. An explanation for this difference is the use of a smaller tip for AFM indentation experiment in our study. Several previous studies also reported that the elastic modulus of cell obtained by using nano-size tip was always higher than those using micro-size spherical tip [28,29]. When a larger tip is used in the cell indentation experiment, subcellular components, including the nucleus and cytoskeleton, share and dissipate the loading imposed. This phenomenon can also be explained by the fact that the cortical actin cytoskeleton in cell membrane is stiffer than the underlying cytoskeleton. The nano-size tip is more sensitive in probing the localized mechanical response induced by the cytoskeleton, even the indentation depth is shallow within sub-microscale.

Consistent with the literature, it is found that the higher loading rate would result in larger value of $C_{10}$, which is proportion to elastic modulus with function of $E=6C_{10}$. The trend of $D_{1}$ is opposite to $C_{10}$ under this circumstance. It is interesting to note that the variation of and $D_{1}$ is less than 50%, while the load rates varied by 20 times (0.1 μm/s to 2 μm/s). The other two parameters exhibit irregular regulation to the indentation rate, which results in a fluctuation of the results of $g_{1}$ and $\tau_{1}$. It is different from a previous study that the value of $g_{1}$ and $\tau_{1}$ were stable with the variation of indentation rate in this work [30].

The lowest standard deviation on material properties is $C_{10}$, which is directly correlated to elastic modulus of bEnd.3 cell. A smaller standard deviation of elastic modulus than previous investigation indicates that the approach in this work is able to precisely characterize the elastic properties of bEnd.3 cell. The material constant $D_{1}$, which is inversely proportional to elastic modulus, exhibits the second lowest standard deviation. Both $g_{1}$ and $\tau_{1}$ disperse significantly more than the other two parameters, which is due to viscoelastic properties being more sensitive to the variation of loading rate. It is important to note that the biological variations of cells are also part of the contribution of the standard deviations.

In this study, cell nanoindentation experimentation was performed in a fluid environment, which alleviates the effects of adhesion and friction. For this reason, surface interactions, including adhesion and friction between cell, tip, and substrate, are not considered in this work. However, adhesion between tip and cell is inevitable, so further investigations should implement the Johnson–Kendall–Roberts (JKR) model to describe the interaction between tip and cell. Another important issue is the morphological variation of cell components such as cytoskeleton under different loading profile. Future study is necessary to carry out more sophisticated works combining a high-resolution (confocal) imaging technique with AFM nanoindentation experiments of cells described by a comprehensive constitutive model.

## 5. Conclusions

This study is a pioneer work to describe the mechanical response of bEnd.3 cells by a viscohyperelastic model under different loading rates. Mechanical properties of cells were determined by a hybrid approach combined AFM nanoindentation experiment and optimized inverse finite element analysis. The hyperelastic properties were described by a Neo-Hookean model and viscoelastic properties were described by a Prony series function. The mechanical characterization framework presented in this work is able to separate the rate-independent and viscous related properties, which were identified as inherent characteristic of vascular endothelial cells. Four parameters were obtained regarding different loading rates by implementing this approach, which is able to precisely determine the value of these parameters. Short-term peak force exhibits a more sensitive reaction to the effect of loading rate than long-term force. Mechanical parameters of $C_{10}$ and $D_{1}$ show a regular relation to the variation of loading rate, while $g_{1}$ and $\tau_{1}$ exhibit an irregular order. This study will provide useful information to expand the understanding of the interaction between vascular endothelial cells and nanoparticles that may enter into the brain tissue.

## Figures and Tables

**Table 1:** The ranges of material parameters used in the parametric analysis.

Material Parameters	Range
$C_{10}$ (kPa)	1–4
$D_{1}$ (kPa^−1^)	0–0.5
$g_{1}$	0.2–0.8
$\tau_{1}$ (s)	0.1–5

**Table 2:** Mechanical properties of bEnd.3 cells determined by using the optimized procedure under different loading rates.

M Indentation Rate (μm/s)	E (kPa)	$C_{10}\;(\mathrm{kPa})$	$D_{1}\;({\mathrm{kPa}}^{-1})$	$g_{1}$	$\tau_{1}\;(s)$
0.1	16.08 ± 1.38	2.68 ± 0.23	0.42 ± 0.05	0.38 ± 0.17	2.55 ± 1.05
0.5	18.42 ± 1.08	3.07 ± 0.18	0.39 ± 0.10	0.45 ± 0.21	1.27 ± 0.85
1	18.90 ± 0.84	3.15 ± 0.14	0.37 ± 0.08	0.47 ± 0.16	1.85 ± 0.95
2	19.92 ± 1.92	3.32 ± 0.32	0.34 ± 0.11	0.35 ± 0.15	3.25 ± 1.11
